# Investigating the Mechanical Behaviour of Viscoelastic and Brittle Pharmaceutical Excipients During Tabletting: Revealing the Unobvious Potential of Advanced Compaction Simulation

**DOI:** 10.3390/pharmaceutics17121606

**Published:** 2025-12-13

**Authors:** Daniel Zakowiecki, Kirils Kukuls, Krzysztof Cal, Adrien Pelloux, Valentyn Mohylyuk

**Affiliations:** 1 Chemische Fabrik Budenheim KG, Rheinstrasse 27, 55257 Budenheim, Germany; daniel.zakowiecki@budenheim.com; 2 Leading Research Group, Faculty of Pharmacy, Rīga Stradiņš University, Konsula Str. 21, LV-1007 Riga, Latvia; 3 Department of Pharmaceutical Technology, Faculty of Farmacy, Medical University of Gdansk, Al. Gen. J. Hallera 107, 80-416 Gdansk, Poland; 4 Application Laboratory, Medelpharm S.A.S, 01700 Beynost, France; apelloux@medelpharm.com

**Keywords:** tabletting, compaction simulation, excipients, anhydrous dibasic calcium phosphate, microcrystalline cellulose, viscoelastic deformation, brittle fracture

## Abstract

**Background**: The compaction of formulation blends is a critical stage in pharmaceutical tablet manufacturing, particularly when drug substances or functional excipients exhibit limited flowability and tabletability. **Objectives**: This study systematically examined the mechanical behaviour of viscoelastic microcrystalline cellulose (MCC) and brittle anhydrous dibasic calcium phosphate (DCPA), as well as their mixtures, to check how deformation mechanisms influence powder handling and tablet performance. **Methods**: A compaction simulator, mimicking a small rotary tablet press, was used to evaluate tablet weight variability, densification profiles, die-filling height, force–displacement behaviour, and in-die Heckel analysis. Additional assessments included compression times, breaking force, tensile strength, elastic recovery, as well as in-die and out-of-die tablet thickness across various compositions and compaction pressures. **Results/Conclusions**: Bulk density values from the simulator showed strong correlation with pharmacopeial measurements (R^2^ ≥ 0.997). Measurable differences in true density and cohesiveness led to poor flowability for MCC and good flow for DCPA, with mixtures containing higher DCPA concentration displaying markedly improved flow characteristic. Compaction analyses confirmed extensive plastic deformation for MCC and fragmentation for DCPA. Increasing MCC content elevated die-fill height, compaction energy, and tablet weight variability, whereas higher DCPA fractions decreased apparent density of tablets and reduced energy demand. Tabletability and compressibility profiles reflected that MCC generated hard tablets but exhibited higher elastic recovery, while DCPA formed softer tablets with closer to linear strength–pressure relationships. Energy profiling demonstrated that MCC stored more elastic energy and required higher overall compression work, whereas DCPA reduced elastic accumulation. Overall, blending viscoelastic and brittle excipients offers a robust strategy for optimizing manufacturability, mechanical strength, and energy efficiency in tablet production.

## 1. Introduction

For many years, compressed tablets have been the most widely used dosage form, offering high patient acceptability, a well-understood manufacturing process, and cost-effectiveness [1,2]. Recently, drug manufacturers have increasingly adopted direct compression (DC) to reduce production costs because it eliminates granulation, which is a time- and energy intensive process. DC is also well suited for heat- or moisture sensitive active pharmaceutical ingredients (APIs). Although it involves the fewest processing steps, DC is very sensitive to variations in API properties and performs suboptimal with poorly flowing materials [3,4].

The vast majority of drug substances, as well as functional excipients, have limited flowability and compactibility. Ensuring suitable powder flowability is particularly important, as in production lines they pass through numerous pipes and pieces of equipment. Mixtures of powders with differing flow properties can segregate, while insufficient flow may cause blockages that stop the process. In the tablet press, poorly flowing mixtures fill matrices unevenly, leading to high variability in tablet weight and non-uniform dosage. Poor tabletting properties can also reduce mechanical strength of the tablets, so they may be damaged or even break during subsequent process steps, including coating or packaging [5,6,7,8,9,10]. Overcoming these issues is crucial for efficient tabletting, particularly with DC technology. Proper selection of specialized DC-grade excipients helps counteract the adverse properties of drug substances and ensures tablets with the required mechanical strength [11]. Excipients used in large quantities to enhance production and performance are known as fillers-diluents (or fillers-binders) [12,13,14]. They can be further divided into three types based on their compression behaviour: brittle, viscoelastic (ductile), and elastic. During tableting, brittle substances such as lactose, dibasic calcium phosphate, or mannitol fracture first, and, if further fragmentation is impossible, may then deform by yielding. Ductile materials like microcrystalline cellulose show significant plastic (irreversible) and elastic (reversible) deformation, whereas elastic materials such as starches deform predominantly reversibly [14,15]. Thorough knowledge of the deformation behaviour of individual substances and their blends is essential for designing an efficient tablet manufacturing with high-speed rotary presses that produce tablets of the desired quality [16].

The aim of the present study was to evaluate the potential offered by a compaction simulator for examining the deformation behaviour of selected excipients commonly used in tablet formulations. Employment of compression simulation during formulation and process development helps reduce scale-up failures by identifying defects such as capping and lamination at an early stage. Because only limited quantities of drug substances are typically available during development, and their cost is often high, the use of compaction simulators can facilitate rapid formulation development while minimizing material consumption.

**Figure 1:**
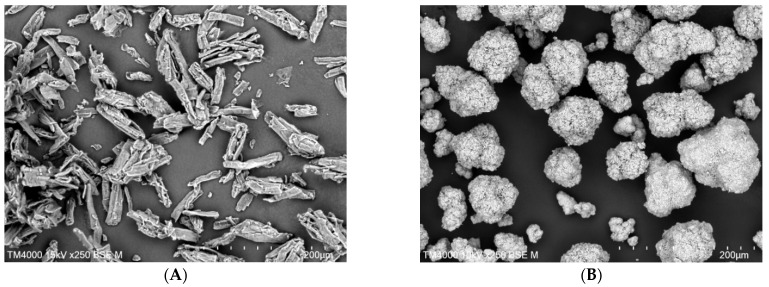
SEM micrographs of (**A**) MCC Ceolus™ UF-711 and (**B**) DCPA PharSQ^®^ Coarse A 60 powders (magnification: ×250).

Viscoelastically deforming microcrystalline cellulose (MCC), brittle anhydrous dibasic calcium phosphate (DCPA), and their mixtures were investigated in this study. The true densities of these two materials are 1.512–1.668 g/cm^3^ [17] and 2.89 g/cm^3^ [18], respectively. From the many commercially available grades, two types with comparable average particle sizes but significant differences in bulk density, porosity, and particle shape were selected for the study. MCC, Ceolus™ UF-711 (Asahi Kasei, Tokyo, Japan), has irregularly shaped fibrous particles with an average particle size of approximately 50 μm (the particle morphology is shown in Figure 1A). It is characterized by high porosity and a low bulk density of about 0.22 g/cm^3^, as stated by the manufacturer [19]. In contrast, particles of DCPA PharSQ^®^ Coarse A 60 (Chemische Fabrik Budenheim KG, Budenheim, Germany) are densely packed agglomerates with fairly spherical shapes, averaging around 60 μm in size (the particle morphology is shown in Figure 1B). The powder exhibits a relatively high bulk density of approximately 1.3 g/cm^3^ and very low porosity [20].

The study analysed changes in the compaction behaviour of powders during tabletting as their nature shifted from viscoelastic to brittle. A compaction simulator was used to precisely assess deformation and densification, based on force–displacement and tabletability profiles. The research presented here continued earlier work. In these works, using the same formulations, in-die/out-of-die Heckel plots and brittle deformation of DCPA, as well as dwell time according to force as a function of powder composition and compression force, were investigated [21,22].

**Figure 2:**
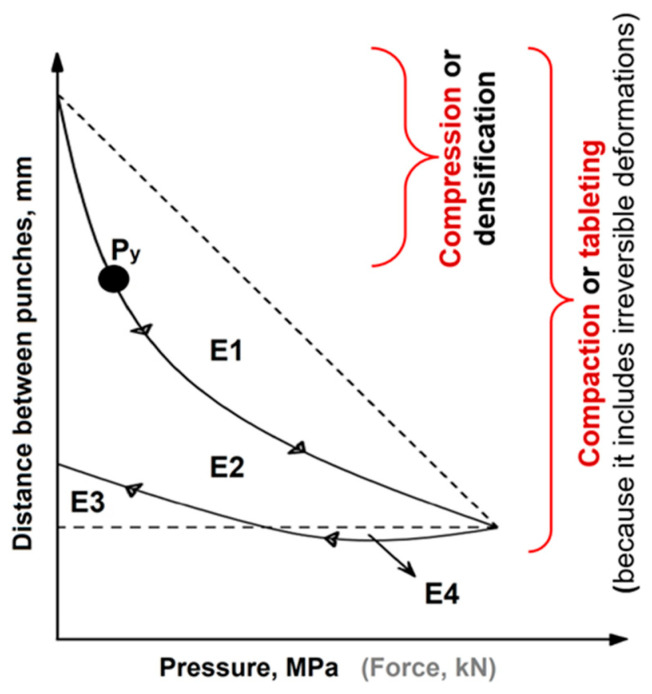
Schematic force–displacement profile with indicated energy distribution during various compression stages (adopted from [23]).

Figure 2 shows an example of a force–displacement plot illustrating the stages of powder densification during tabletting and energy distribution.

E1 represents rearrangement energy. The sum of E2 and E4 is the plastic energy, the total energy provided to the tablet during compaction. E3 is the elastic energy (or energy lost), as the product returns energy by pushing the punches back. E4 represents plastic flow energy, which occurs when the force decreases while the material continues to deform. This phenomenon is characteristic of ductile materials. E4 arises because the compression pressure remains higher than the mean yield pressure (Py). The sum of E1, E2, and E3 constitutes the compaction energy. Py is a measure of plasticity, indicating the point beyond which deformations become irreversible. It should be noted that, although yield typically occurs over a range, the Py value derived from a Heckel plot is treated as a single point representing the average yield pressure.

In the study, a STYL’One Nano compaction simulator (Medelpharm, Beynost, France) was used to mimic the compression dynamics of small industrial rotary tablet presses at 70 rpm and compaction forces of 10–50 kN (equivalent to 100–500 MPa). With high-accuracy sensors, it enabled detailed monitoring of tabletting process, providing insights into elastic recovery, elasticity, compression energy, plastic energy, elastic energy, rearrangement energy, specific work of compression, and cohesion index [7,24,25]. Comprehensive analysis of compaction behaviour with a compression simulator can help anticipate production issues and prevent defects associated with over-compression.

## 2. Materials and Methods

### 2.1. Materials

Microcrystalline cellulose (MCC), CEOLUS^TM^ UF-711 (Asahi Kasei, Tokyo, Japan), shown in Figure 1A. Anhydrous dibasic calcium phosphate (DCPA, CaHPO_4_), PharSQ^®^ Coarse A 60 (Chemische Fabrik Budenheim KG, Budenheim, Germany), shown in Figure 1B. Precipitated amorphous silica SYLOID^®^ 244 FP (Grace GmbH, Worms, Germany). Sodium stearyl fumarate (SSF) PRUV^®^ (JRS Pharma, Rosenberg, Germany).

### 2.2. Preparation of Tablet Blends and Assessment of Their Properties

Powder mixtures described in Table 1 were prepared in a multistep procedure. First, MCC, DCPA, and silica were mixed for 10 min in a double-cone blender (DVC Developer, Comasa, Barcelona, Spain).

The obtained mixtures were then sieved through a 1.0 mm sieve and mixed again for another 5 min. SSF was sieved through a 0.5 mm sieve, added to the blends containing MCC, DCPA, and silica, and the entire powders were mixed for 2 min. The obtained blends were sieved through a 1.0 mm sieve and mixed again for 2 min. Formulation codes (F 100-0, F 75-25, etc.) reflect the volume ratio of MCC to DCPA. The volume ratio, excluding the porosity of the materials, was calculated based on the true density of the components.

#### 2.2.1. Determination of True, Bulk and Tapped Densities

The true density (*ρ_t_*) of the tablet compositions shown in Table 1 was calculated by taking into account the weight shares of individual components (*x_comp_*) and their true densities (*ρ*_comp_): 1.59 g/cm^3^ for MCC [17], 2.89 g/cm^3^ for DCPA [18], 1.11 g/cm^3^ for sodium stearyl fumarate [26], and 2.20 g/cm^3^ for silicon dioxide [27], using the following equation [21,28,29]:

$$\rho_{t}=\left(\rho_{comp1}\cdot x_{comp1}\right)+\ldots +\left(\rho_{comp\;i}\cdot x_{compi}\right)$$

The prepared tablet blends with the compositions shown in Table 1 were analysed in terms of their bulk and tapped density according to the description for Method 1 “Measurement in a graduated cylinder” provided in accordance with the European Pharmacopoeia, using a SVM II Tapped Density Tester (Erweka GmbH, Langen, Germany). When measuring the tapped density, the number of taps was varied, and readings were taken after 10, 110, 1110, and 3110 taps. All measurements were performed in triplicate (n = 3).

Additionally, bulk density was determined based on the preset position of the lower punch (filling depth) in the compaction simulator. It was calculated by dividing the mass of the die content (tablet mass) by the volume of the filled die, which was based on the volume of the cylindrical geometry using the known internal diameter of the die and the filling depth.

#### 2.2.2. Characterization of Powder Flow Properties

Based on the results from the powder density measurements, the flowability of the powders was characterized by calculating the Carr index and Hausner ratio, as described in European Pharmacopoeia.

The mass flow rate of the powder blends was determined according to European Pharmacopoeia using a GTB flow tester (Erweka GmbH, Langen, Germany). A 100 g sample was placed in a stainless-steel cone funnel with a closed bottom. After opening the orifice, the time taken for the sample to flow out was automatically measured. The mass flow rate was calculated based on the time required for the powder to flow through orifices with diameters of 10, 15, and 25 mm and expressed in g/s. All measurements were performed in triplicate (n = 3).

### 2.3. Preparation of Tablets

Powder blends were compressed using the compaction equipped with flat-faced 11.28 mm diameter round punches to achieve a target mass of 500 mg. The compression cycles simulated a small rotary tablet press with a turret diameter of 180 mm, a pre-compression roll diameter of 44 mm, and an angle between rollers of 65 degrees. The compression roll diameter was 160 mm, with a 60-degree angle between the main compression and the beginning of the compression ramp, and a 20-degree ejection ramp angle. Tableting speed was set to 70 rpm (the simulator’s maximum). Pre-compaction force was 5 kN (50 MPa), and compaction forces ranged from 10 to 50 kN (100 to 500 MPa). Powder feeding into the die was automated using a feed shoe [21,22]. Raw data is available [30].

### 2.4. Measurement of Tablet Hardness and Calculation of Tensile Strength

The tablet dimensions and breaking force (hardness) were measured in accordance with European Pharmacopoeia for 10 tablets (n = 10) using the ST50 WTDH tablet testing system (SOTAX AG, Aesch, Switzerland) immediately after tableting (out-of-die measurements). The radial tensile strength (*σ*x) was calculated using the following equation:

$$\sigma_{x}=\frac{2F}{\pi Dh}$$

where *F* is the tablet breaking force (crushing strength), and *D* and *h* represent the tablet’s diameter and height (thickness), respectively [31].

### 2.5. Characterization of the Tabletting Process

Force-displacement data, along with plastic energy (J), elastic energy (J), compression energy (J), rearrangement energy (J), and ejection energy (J), were automatically recorded using the STYL’One Nano compaction simulator (Medelpharm, Beynost, France).

To calculate the specific work of compression (J/g), the compression energy (J) was divided by the weight of the resulting tablet (g) [32].

The cohesion index (MPa/J) was estimated by dividing the tensile strength (MPa) of the tablets by the corresponding compression energy (J) [33].

The elasticity (%) was calculated using the following equation:

$$Elasticity\;\left(\%\right)=\;\frac{Elastic\;energy\;\left(J\right)}{Compression\;energy\;\left(J\right)}\cdot \;100\%$$

### 2.6. Calculation of Apparent Density, Porosity, and Solid Fraction (Out-of-Die Method)

The apparent density (*ρ_a_*) of the tablets was determined after ejection from the die (out-die method). Tablets were prepared using flat-faced punches, so they had a cylindrical shape. Therefore, *ρ*_a_ was calculated as the ratio of tablet mass (*m_tab_*) to the volume of the cylinder using the following equation:

$$\rho_{a}=\frac{m_{tab}}{\pi {\cdot \left(\frac{D}{2}\right)}^{2}\cdot h}$$

where *D* and *h* represent the tablet’s diameter and height (thickness), respectively.

The solid fraction (*SF*) and porosity (*ε*) of the tablets were calculated based on USP42-NF37 Chapter <1062> “Tablet compression characterization” using the following equation:

$$SF=\frac{\rho_{a}}{\rho_{t}}=(1-\varepsilon )$$

The porosity of the tablets was quantified as the inverse of the solid fraction (*ε = 1 − SF*) [34,35,36].

### 2.7. Evaluation of Compaction Behaviour of Tablet Blends (The Compaction Triangle)

Following the description provided in USP42–NF37 Chapter <1062> “Tablet Compression Characterization”, three relationships between tableting parameters, namely tabletability, compactability, and compressibility, collectively referred to as the compaction triangle, were employed to assess the compaction behaviour of the tablet blends. Along with the manufacturability profile, they were determined based on the obtained data including tablet hardness, tablet tensile strength, ejected solid fraction, and compression force (or compaction pressure) [36].

### 2.8. Elastic Recovery

The elastic recovery (ER) was estimated as a percentage of tablet recovery based on the tablet height (thickness) after ejection (*h*_out-of-die_) and the in-die tablet height at the minimal punch separation (*h*_in-die_), using the following equation [37]:

$$ER=\frac{h_{out-of-die}-h_{in-die}}{h_{in-die}}\cdot 100\%$$

### 2.9. Visualisation of Tablet Size and Shape

TaBlitz™ Pharmaceutical Tablet Design Software (accessed in 2025.07.10 via https://tablitz.app/; St. Louis, MO, USA) [38] was employed to visualize tablet size and shape across various punch formats. The calculations were based on data obtained during this study. Results are shown for formulations demonstrating distinct deformation behaviours during compaction: viscoelastic (F 100-0), brittle (F 0-100), and intermediate (F 50-50), with compositions are summarized in Table 1.

## 3. Results and Discussion

### 3.1. Characteristics of Powder Properties

**Figure 3:**
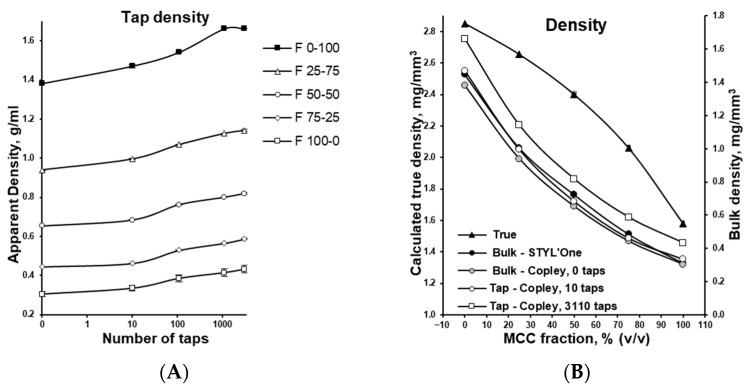
Characteristics of powder mixtures with varying MCC and DCPA ratios: density changes during tapping (**A**), comparison of bulk, tapped, and true densities (**B**).

The study investigated viscoelastically deforming MCC, brittle DCPA, and their mixtures, as summarized in Table 1. The two materials differed markedly in bulk density (see Figure 3A), influenced not only by their distinctly different true densities (see Figure 3B), but also particle shapes (see Figure 1). Tapping caused rapid particle settling and rearrangement, filling voids between them and displacing the gaseous phase. This process can be considered comparable to the particle rearrangement that occurs during the initial phase of tableting. After approximately 1000 taps, the process stabilized, and further densification of the powder bed was minimal. However, the large difference between the tapped density (after 3110 taps) and the true density indicates that a substantial amount of gaseous phase remained entrapped between particles and partially retained within their internal pores. Figure 3B compares bulk densities of powders with varying MCC/DCPA ratios, measured by both a graduated cylinder and the compaction simulator, which gave nearly identical values. This indicates that, in some cases, bulk density can be directly assessed with minimal material using a compaction simulator.

**Figure 4:**
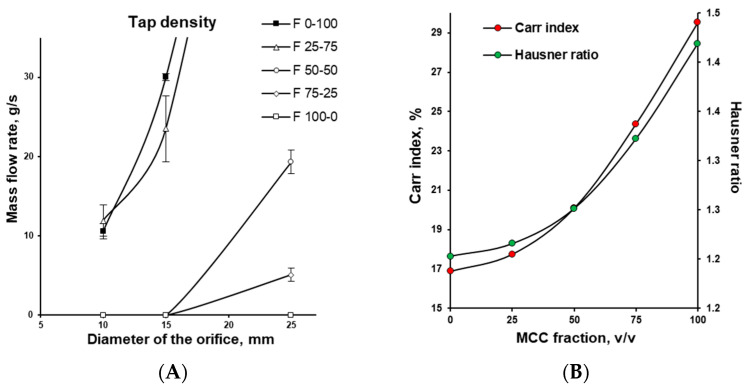
Flowability of powder mixtures with varying MCC and DCPA ratios: mass flow rate (**A**), Carr index and Hausner ratio (**B**).

Differences in particle structure and material density measurably affected the flow characteristic of examined powders (see Figure 4). Used type of MCC showed poor flowability, with flow through even large-diameter orifices being completely inhibited. This behaviour was driven by particle morphology, low density, and cohesion forces, which caused MCC particles to attract and form bridges blocking the orifice. In contrast, DCPA, composed of more spherical, denser particles, exhibited unconfined flow. Even with a narrow orifice (below 10 mm), mass flow rates were high and increased sharply with larger orifice diameters (above 15 mm), markedly exceeding 50 g/s. In the mixtures, increasing DCPA content significantly improved powder flow (see Figure 4A). As illustrated in Figure 4B, mixtures with a lower concentration of MCC (less than approximately 50% *v*/*v*) demonstrated substantially better flow, reflected in pronounced improvements in Carr Index and Hausner Ratio.

### 3.2. Tablet Compression Characterization

**Figure 5:**
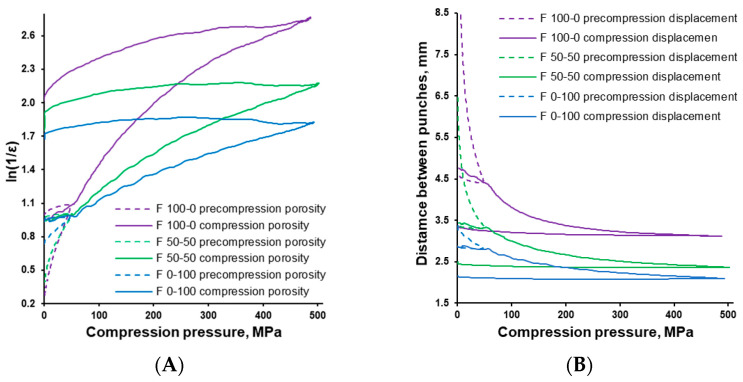
In-die Heckel plots (**A**) and force-displacement profiles (**B**) recorded during compression of a viscoelastic formulation (F 100-0), a brittle formulation (F 0-100), and an intermediate formulation (F 50-50). Dotted lines represent the pre-compression phase, while solid lines indicate the main compression stage.

The compaction behaviour of the tested powder mixtures was evaluated using the Heckel plot and force-displacement profile shown in Figure 5. These overlaid plots illustrate tablet porosity {ln(1/ɛ)} and punch displacement (mm) as functions of the applied compaction pressure. For clarity, only three formulations are shown: viscoelastic (F 100-0), brittle (F 0-100), and intermediate (F 50-50). Each exhibited distinct deformation behaviour, illustrating the trend in compaction as the proportion of materials with different compression characteristics varied. Although only these three formulations are presented here, the subsequent discussion encompasses all tablet mixtures investigated.

**Figure 6:**
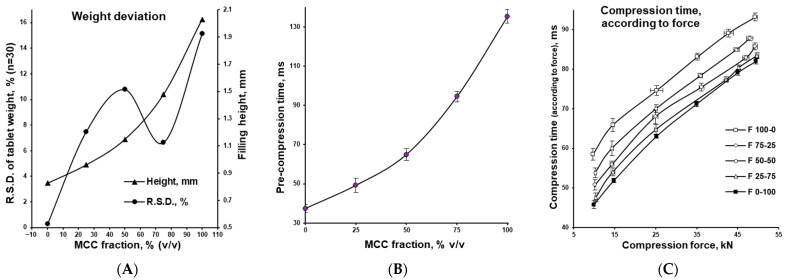
Effect of changing MCC/DCPA ratio on filling height and tablet weight variation (**A**), pre-compression time (**B**) and main compression time for the tablets compressed under different compaction pressures (**C**).

Figure 6 summarizes the compression behaviour of the tableting mixtures listed in Table 1, as determined with the compaction simulator based on the analyses in Figure 5. Plot A shows the effect of the MCC/DCPA ratio on die filling and tablet weight variability, demonstrating how powder density influences filling height and, consequently, the consistency of tablet weights, expressed as the relative standard deviation (RSD) of tablet masses. Plot B illustrates the change in the time required to reach a pre-compression force of 5 kN (50 MPa) as a function of the MCC/DCPA ratio. Plot C compares the time needed to reach the target main compression force (10–50 kN) for mixtures with different MCC/DCPA ratios.

MCC, due to its much lower density compared with DCPA (see Figure 3), occupied a larger volume in the tablet press dies (see the filling height in Figure 6A). Under applied compression force, it densified to a greater extent than DCPA (see Figure 5A) but required deeper punch penetration into the die (see Figure 5B). This process was quantified by measuring the time needed for the punch to reach the preset compression force (see Figure 6B,C). During pre-compression, MCC required substantially more time for particle rearrangement, partial air removal, and compaction to a porosity level comparable to DCPA (see Figure 6B). In the main compression phase, time differences were smaller but remained significant.

Overall, powder densification (see Figure 5A), compaction energy (area under the force–displacement curve in Figure 5B), die filling height, and both pre- and main compression times (see Figure 6A–C) were directly proportional to the MCC/DCPA ratio. It was also worth noting that a deterioration in the flowability of powders, along with an increased volume of powder filled into the dies, negatively affected tablet weight variation (see Figure 6A), potentially compromising the uniformity of dosage units. Such data are valuable in tablet development for optimizing geometry (e.g., diameter-to-height ratio), improving coating and packaging suitability, and enhancing overall product quality [39,40,41].

**Figure 7:**
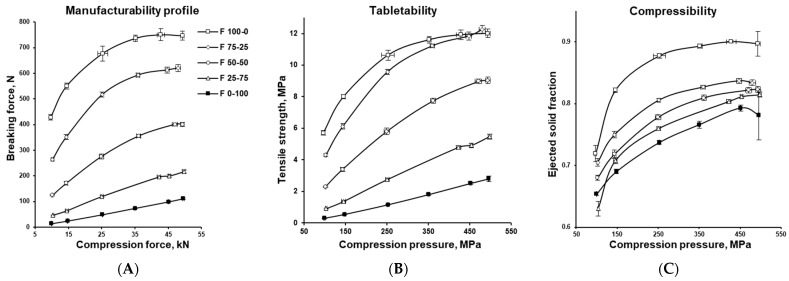
Characteristics of tabletting process: manufacturability (**A**), tabletability (**B**), and compressibility profile (**C**).

Tablet compression profiles, as described in USP42–NF37 Chapter <1062>, were used to investigate the behaviour of the studied blends during tableting, specifically, their response to applied pressure, compaction behaviour, and susceptibility to defects such as lamination or capping. The manufacturability, tabletability, and compressibility profiles shown in Figure 7A–C provide a comprehensive assessment of formulation performance, illustrating how tablet mechanical strength changes with increasing compression force (compaction pressure), indicating overall suitability for tablet production.

Plot A shows the manufacturability profile, where tablet hardness (breaking force) is plotted against compaction force, reflecting the practical suitability of a powder for tablet manufacturing. Plot B presents the tabletability profile, which plots tensile strength against compaction pressure to evaluate whether a powder can be compressed into tablets of adequate strength. While these graphs appear similar, the manufacturability curve is more relevant for setting production parameters to achieve the desired product quality, whereas the tabletability curve is particularly useful for comparing formulations with markedly different properties (e.g., density). By accounting for tablet dimensions (see tensile strength equation in Section 2.4), tabletability offsets the influence of factors such as thickness and diameter.

MCC showed excellent tableting properties due to its plastic deformation behaviour and low Py. With increasing compaction pressure, it densified rapidly, reaching a high solid fraction (see Figure 7C). In this state, densely packed particles formed numerous interparticulate contact points, and strong cohesion through hydrogen bonding and mechanical interlocking enhanced bonding, producing tablets with very high mechanical strength (see Figure 7A,B) [42,43]. A characteristic feature of this viscoelastic material was the plateau region, beyond which further increases in compression force no longer improved densification or tablet hardness (breaking force or tensile strength). By contrast, DCPA exhibited a linear increase in tablet mechanical strength with rising compaction pressure, a behaviour typical of brittle materials. During compression, fragmentation of DCPA generated many new clean surfaces that promoted interparticle bonding, making brittle substances generally less sensitive to lubrication and tableting speed [44,45].

**Figure 8:**
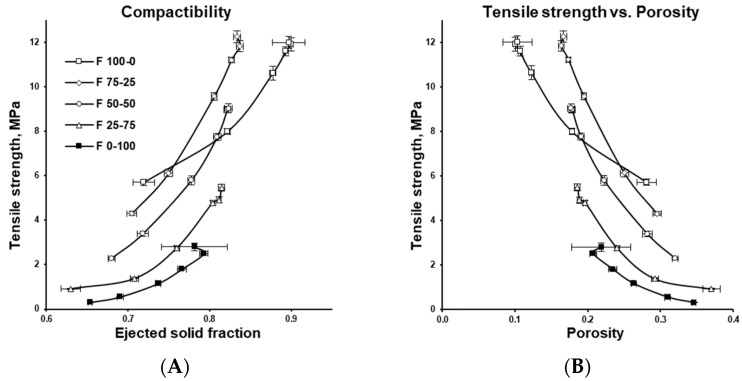
Compactibility profiles of the investigated formulations: the relationship between tablet tensile strength and ejected solid fraction (**A**) or tablet porosity (**B**).

Figure 8 presents relationship between tablet tensile strength and either ejected (out-of-die) solid fraction or porosity. Solid fraction, calculated as the ratio of tablet apparent density to the true density of its components (see Section 2.2.1), represents the proportion of solid matter in the tablet. Porosity, its complement, is obtained by subtracting the solid fraction from 1 (porosity = 1 − solid fraction). The compactability profile is independent of tableting speed, serves as a predictor of tablet mechanical strength in both formulation development and process scale-up.

Tablet mechanical strength (tensile strength) increased exponentially with solid fraction, likely due to dipole–dipole interactions [46,47], while porosity decreased proportionally (see Figure 8A,B). Lower solid fractions (higher porosity), as seen for DCPA, produced softer tablets, whereas high solid fractions (low porosity) of MCC resulted in very hard tablets (compare Figure 7A–C). Excessive compaction at high solid fractions, however, led to over-compression, evident in the drop-off at the end of MCC’s manufacturability, tabletability, and compressibility profiles. For DCPA, over-compression was apparent only in the compressibility profile. In both cases, this was accompanied by high variability caused by defects such as microcracks, fractures, and chipping. Very high solid fractions may also impair tablet disintegration and slow drug dissolution [48,49].

Balancing the properties of formulation components is therefore essential for optimizing overall tablet performance. Using excipients with different deformation mechanisms in varying proportions can create a synergistic effect, enhancing advantages while minimizing drawbacks.

### 3.3. Energetic Characterization of the Tableting Process

**Figure 9:**
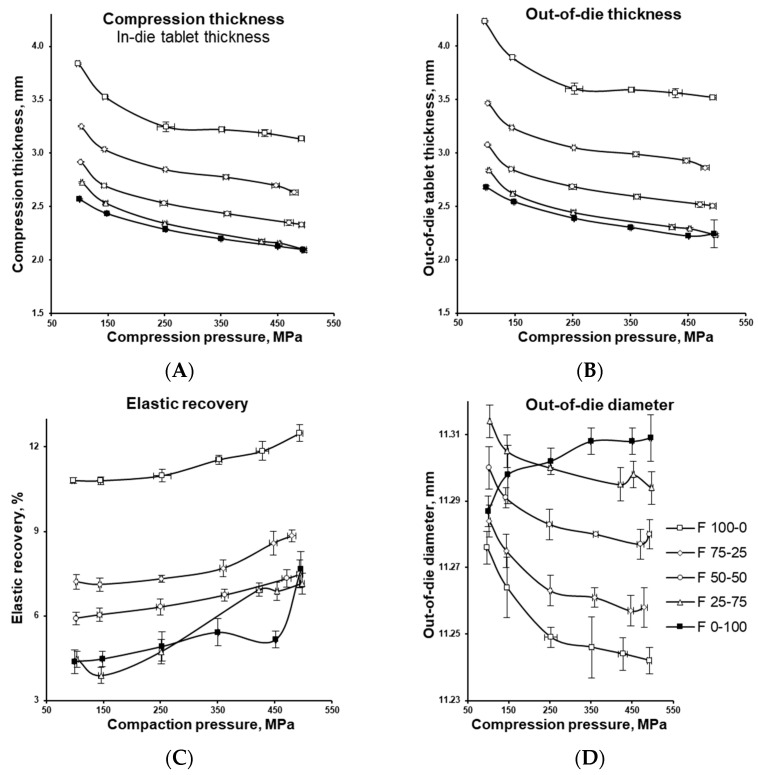
Thickness of the tablets with varying MCC and DCPA ratios, compressed under different compaction pressures, as measured in-die (**A**), immediately after ejection (**B**), and the difference between in-die and out-of-die thickness (**C**). The out-of-die diameter as measured immediately after ejection (**D**).

Figure 9 illustrate the elastic relaxation behaviour of tablets with varying MCC/DCPA ratios compressed under different compaction forces. This is shown by comparing tablet thickness measured in-die and immediately after ejection (Figure 9A,B). Since the in-die diameter is fixed at 11.28 mm (see Section 2.3), Figure 9D presents tablet diameters measured after ejection.

**Figure 10:**
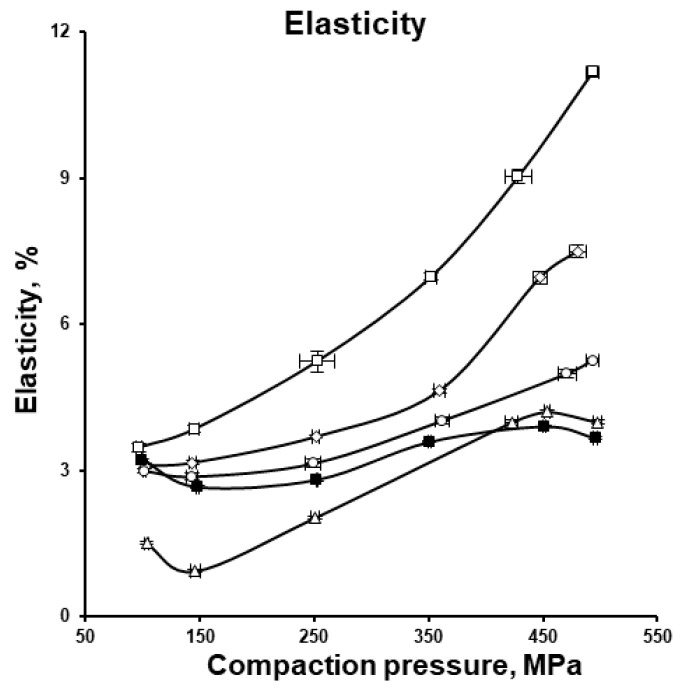
Elasticity in % of the tablets with varying MCC and DCPA ratio, compressed under different compaction pressures.

Being the ratio between compression and elastic energy expressed in percentage, elasticity can be used to show the difference in formulations in their ability to convert the overall applied energy into energy spent for the elastic recovery (Figure 10). Although irreversible plastic deformation, including brittle fragmentation, predominates during the compaction of MCC and DCPA and favours tablet formation, elastic deformation also plays a key role. During compression, elastic energy is stored and subsequently released during decompression. When the force is removed, tablets undergo elastic recovery increasing in volume axially while still in the dies and radially after ejection (see Figure 9) [50,51].

MCC-based tablets were significantly thicker than DCPA tablets (see Figure 9A,B), with thickness increasing further as MCC content rose due to its low density (compare Figure 3). Upon ejection, MCC tablets exhibited rapid elastic relaxation, with thickness increases of up to 12% depending on compression force. The addition of DCPA markedly suppressed this effect, and in DCPA-based formulations the elastic recovery was nearly four times smaller than in MCC tablets (see Figure 9C).

Analysis of tablet diameter changes (Figure 9D) revealed notable differences between the excipients not only in the extent but also in the direction of relaxation, linked to the anisotropic nature of the particles [52,53]. MCC particles preferentially oriented during compression and deformed axially and radially. Upon decompression, they recovered mainly along the axis of compression while contracting radially, causing a slight reduction in diameter after ejection. The magnitude of these deformations increased with applied compression force. In contrast, DCPA particles fragmented during tableting; residual compression energy induced radial stress relaxation of the fragments, resulting in a slight increase in tablet diameter.

**Figure 11:**
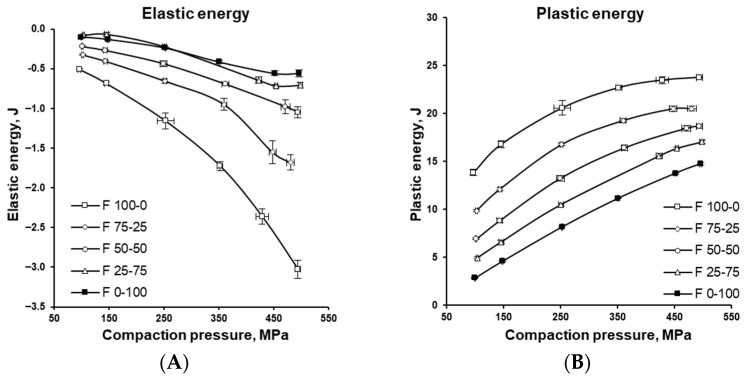
Elastic energy (**A**) and plastic energy (**B**) of the tablets with varying MCC and DCPA ratio, compressed under different compaction pressures.

**Figure 12:**
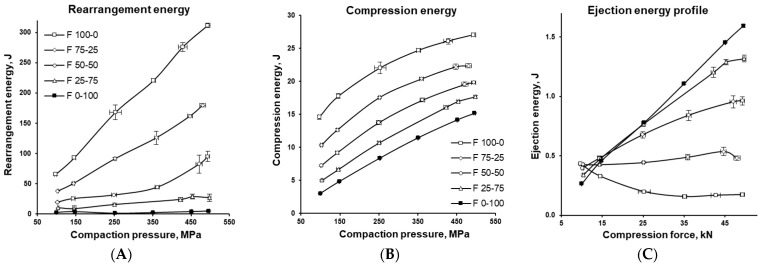
Rearrangement energy (**A**), compression energy (**B**), and ejection energy (**C**) of the tablets with varying MCC and DCPA ratio, compressed under different compaction pressures.

**Figure 13:**
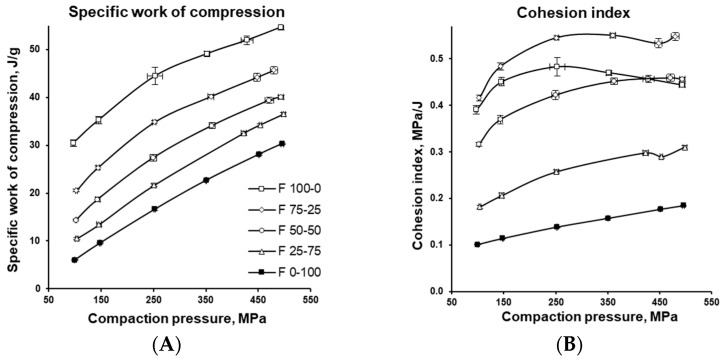
Specific work of compression (**A**) and cohesion index (**B**) of the tablets with varying MCC and DCPA ratio, compressed under different compaction pressures.

Figure 11 and Figure 12 illustrate how the various energies involved in tablet formation (namely elastic, plastic, rearrangement, compression, and ejection energies) vary with the MCC-to-DCPA ratio and increasing compaction pressure, providing insight into the compaction behaviour of the formulations. The negative value of elastic energy does not imply the presence of “negative energy,” but indicates the direction of the process. In tablets, energy is released, which is considered negative relative to the input energy, meaning that energy is recovered rather than stored. Figure 13 further characterizes the formulations through two parameters relevant to tablet production. Plot A shows the specific work of compression, representing the energy in Joules (J) required to compress one gram (g) of the tested formulations into tablets. Plot B shows the Cohesion Index, which reflects intermolecular forces between particles, influencing both tablet mechanical strength and flowability.

The degree of elastic recovery depended on both the applied compaction pressure and material properties. For viscoelastic MCC particles, higher pressure led to greater accumulation of elastic energy (see Figure 11A) and more pronounced post-compaction recovery than in brittle DCPA particles. Strong elastic relaxation has often been associated with reduced mechanical integrity and tablet defects during decompression and ejection [50,54,55]. In mixed formulations, however, DCPA reduced energy accumulation and elastic relaxation, thereby mitigating these risks.

Comparison of Figure 11 and Figure 12, which present changes in plastic and elastic energy as a function of compression pressure (Figure 11A,B), shows how compression energy is distributed between irreversible plastic deformation and reversible elastic deformation [56]. MCC demonstrated a strong tendency to accumulate elastic energy, yet a large portion of the compression energy was also used for plastic deformation, effectively mitigating tablet defects. In contrast, DCPA, a brittle material that fragments during tableting, used little energy for plastic deformation and showed almost no elastic energy accumulation. In mixtures of MCC and DCPA, the latter contributed significantly to reducing unfavourable excess elastic energy storage, confirming earlier findings.

The energy profiles in Figure 12 provide insights into how the formulations behaved during compaction and how this affected tablet quality, providing guidance for adjusting formulation composition or tableting conditions to ensure robust manufacturing. Increasing the proportion of well-flowing DCPA reduced rearrangement energy and improved particle packing (compare with Figure 3). Compression energy (associated with deformation, fragmentation, and bond formation) was higher for MCC, resulting in stronger tablets, as confirmed in Figure 7. In contrast, DCPA required significantly less compression energy, advantageous for production efficiency, but produced weaker tablets; however, combining it with MCC improved strength. The cohesion index, which reflects the mechanical strength of tablets, was highest for MCC-based formulations, confirming their superior bonding capacity and robustness. Evaluating cohesion index alongside compression energy enables the selection of formulations that balance high mechanical strength with relatively low specific work of compression. Such a balance ensures both efficient processing and high product quality.

At the final stage, ejection energy, reflecting friction between the tablet and die walls, was higher for DCPA, indicating a greater need for lubrication. As a brittle material, DCPA is generally insensitive to excessive lubrication, so higher lubricant levels are unlikely to impair tablet mechanical properties [57,58]. This behaviour can be attributed to radial stress relaxation of DCPA fragments, leading to a slight increase in tablet diameter (Figure 10). In contrast, MCC particles primarily relax axially, which reduced friction and consequently lowered ejection energy when its content is increased. (see Figure 12C).

### 3.4. Visualisation of the Tableting Process

The data collected in this study allowed visualization of the relationships between various process parameters. By combining the energies involved in tableting, i.e., rearrangement, compression, and ejection energy (Figure 12), the total energy consumption per tablet (kWh) could be calculated. Considering electricity costs, this provides a preliminary estimate of the costs associated with the tabletting process.

**Figure 14:**
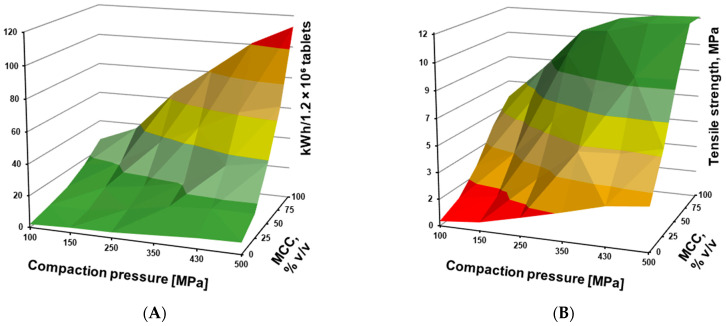
Visualization of the effect of the MCC to DCPA ratio and the applied compaction pressure on the energy consumption during compression of tablet blends into tablets (**A**) and on the mechanical strength of the resulting tablets (**B**).

Figure 14A shows the effect of the MCC-to-DCPA ratio (expressed as MCC fraction, %) and applied compaction pressure on the total energy required to produce 12 million 500 mg tablets. Blends with higher DCPA content required significantly less energy, with only a slight increase at higher compression forces. In contrast, MCC-rich blends consumed substantially more energy, which further increased with higher compression pressures. These values represent a single process estimate, and in large-scale production, where multiple tablet presses operate almost continuously, minimizing energy consumption can offer significant economic benefits and support sustainable development.

Figure 14B summarizes the effect of MCC-to-DCPA ratio and compression force on tablet mechanical strength (tensile strength in MPa), as previously shown in Figure 7. Increasing MCC content markedly enhanced tablet hardness and made the blends more sensitive to applied compaction pressure. It is worth noting that tensile strengths above 1.7 MPa are generally sufficient to withstand industrial processing stresses. On the other hand, excessively high hardness can prolong disintegration time and delay active ingredient release [3,8,59,60,61]. Therefore, it is important to determine the optimal level of mechanical strength for each formulation.

**Figure 15:**
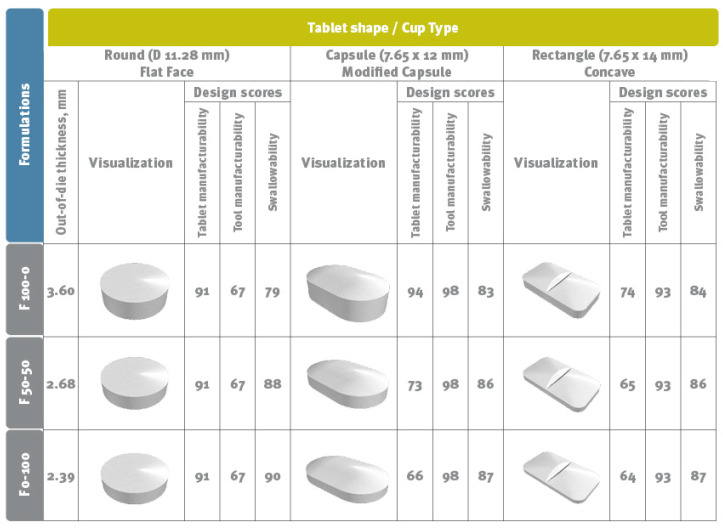
Design of 500 mg tablets: visualization and scoring using TaBliz software.

Visualizing and jointly evaluating relationships between formulation or process variables and quality attributes during pharmaceutical development helps identify balanced parameters for sustainable manufacturing and high-quality tablets. Software tools that simulate tablet appearance and estimate manufacturability and swallowability provide additional support (Figure 15).

Tablet manufacturability considers how well a geometry can be compacted with standard equipment, while tooling manufacturability assesses production efficiency and cost-effectiveness. Swallowability evaluates how tablet shape and size affect ease of swallowing, helping reduce patient discomfort or non-compliance [62,63]. Increasing DCPA content in blends significantly reduces tablet size, improving swallowability due to DCPA’s high density. Conversely, higher MCC content enhances manufacturability through strong intermolecular binding. Regarding tooling and tableting efficiency, both materials exhibit similar characteristics.

## 4. Conclusions

The present study investigated the compaction behaviour of two materials known to exhibit distinct deformation characteristics, namely ductile MCC and brittle DCPA, as well as their mixtures. Among the many different grades available on the market, materials with markedly different properties, including particle morphology and powder density, were selected for this research. A compaction simulator, the STYL’One Nano, was used to simulate a small rotary tablet press. The tableting speed was set to 70 rpm, providing results representative of small-scale production.

The study showed that MCC and DCPA offered different functional properties that contributed to tablet quality attributes in various ways. Formulations based on high-density DCPA were characterized by excellent flowability, which improved tablet weight uniformity. The tablet blends occupied less space in the dies, compaction required less time, and the resulting tablets were smaller in size. However, these tablets exhibited relatively low mechanical integrity. In contrast, formulations based on ductile MCC produced tablets with very high mechanical strength. However, they tended to accumulate excessive elastic energy, resulting in elastic recovery. The combination of the two materials, which differ in their deformation mechanisms, resulted in a synergistic effect so that each component compensated for the other’s deficiencies while enhancing their respective strengths. The ratio of viscoelastic to brittle components in the formulation must reflect the deformation and fragmentation properties of the drug substance to ensure that the resulting tablets possess adequate mechanical strength, are free from defects, and deliver the required performance.

Using a compression simulator yielded data that enabled detailed visualization of the interactions between process parameters and tablet quality attributes, including their simulated appearance. This knowledge facilitates the selection of optimal settings for efficient, sustainable production of high-quality medicinal products.

## Figures and Tables

**Table 1:** Compositions of the tested formulations presented in weight and volume ratios.

Ingredient	F 100-0	F 75-25	F 50-50	F 25-75	F 0-100	F 100-0	F 75-25	F 50-50	F 25-75	F 0-100
	Weight Ratio (% w/w)					Volume Ratio (%)				
MCC	0.977	0.608	0.346	0.151	0.000	96.9	72.3	47.9	23.8	0.0
DCPA	0.000	0.369	0.631	0.826	0.977	0.0	24.1	47.9	71.4	94.6
SSF	0.020	0.020	0.020	0.020	0.020	2.8	3.4	4.0	4.5	5.0
Silica	0.003	0.003	0.003	0.003	0.003	0.2	0.3	0.3	0.3	0.4
**Total**	**1.000**	**1.000**	**1.000**	**1.000**	**1.000**	**100.0**	**100.0**	**100.0**	**100.0**	**100.0**

## Data Availability

The main data used in this manuscript is available in the form of a dataset [30].
